# How Much Treatment are Adolescents Receiving in Specialised Substance Use Healthcare in Sweden? Age and Cohort Trends

**DOI:** 10.1177/29768357251351758

**Published:** 2025-07-04

**Authors:** Patrik Karlsson, Mats Ekendahl, Philip Lindner

**Affiliations:** 1 Department of Social Work, Stockholm University, Sweden; 2 Department of Clinical Neuroscience, Centre for Psychiatry Research, Karolinska Institutet & Stockholm Health Care Services, Sweden; 3 Stockholm Centre for Dependency Disorders, Stockholm Health Care Services, Sweden

**Keywords:** substance use treatment, adolescents, Sweden, Maria Ungdom

## Abstract

**Background:**

There is little systematic research on what adolescents are provided within specialised substance use treatment. This study explored trends in treatment received (number of outpatient treatment visits and inpatient treatment episodes) according to year and age at first contact among adolescents who were enrolled at Sweden’s largest treatment provider, the Maria Ungdom Clinic in Stockholm, between 2011 and 2021.

**Methods:**

The data were derived from electronic health records for all patients (n = 29 967) who were in contact with Maria Ungdom Stockholm between 2011 and 2021. Negative binomial regressions estimated the association between year cohort and age at first contact and number of outpatient visits respective number of inpatient episodes. We also tested whether trends according to year cohort and age at first contact were moderated by sex and treatment type.

**Results:**

Patients in earlier cohorts and those who were younger at first contact had more outpatient visits. A similar association with age at first contact was observed for inpatient treatment, but was less evident for year cohort. Differences in outpatient visits according to year cohort and age at first contact were mainly observed in patients enrolled in both outpatient and inpatient treatment, and the same held true for differences in number of inpatient episodes according to age at first contact. Regression models that adjusted for potential different exposure time found a higher treatment rate among later cohorts and patients who were older at first contact.

**Conclusion:**

Patients in the earlier year cohorts and who were at younger age at first contact with Maria Ungdom received most treatment. However, later cohorts and patients who were at an older age at first contact with Maria Ungdom had a higher treatment rate, suggesting that these groups were provided with a more intensive treatment.

## Introduction

Adolescent substance use (ASU) is a high-priority issue in most countries, and particularly so in Sweden and other countries with strict policies related to ASU. In Sweden, a large number of young people are in contact with ASU treatment facilities. Treatment is often provided at so-called Maria clinics, staffed with both medical and psychosocial professionals and located in municipalities across the country. While adolescents may come into contact with these services for various reasons, a common one is having being caught using substances that are illicit in Sweden, in particular cannabis.^1^ Treatment contact is often initiated by social or juridical authorities, and often not by adolescents or their personal networks,^2^ and ASU treatment in Sweden includes a heterogenous group of patients,^3^ ranging from adolescents who have tried illicit substances for the first time to polydrug users. The patient population is thus likely to include patients with different treatment trajectories,^4^ with some receiving more services than others. Estimates suggest that among those who initiate treatment at the Maria clinics, the majority has cannabis as their “primary” drug and this has increased over time from slightly more than 60% in 2010 to slightly less than 80% in 2021.^2^ Alcohol is the primary drug among a minority and the proportion having alcohol as their primary substance has decreased from about 30% in 2010 to about 10% in 2021. While the proportion who have received prior substance use treatment has been relatively stable over time, there has been a clear upward trend in the proportion with ongoing psychiatric care. Overall, the gender distribution has been quite stable (ranging from 70% to 77% males between 2013 and 2021) and the average age has been steady at 17 years.^2^

However, Sweden’s largest regional, rather than municipal, ASU treatment provider – Maria Ungdom (MU) Stockholm – is not fully covered in these estimates, and geographical differences in the supply of services and in the collaboration between the social services and the regions entail that these estimates may not reflect the situation among a sizeable proportion of adolescents who are engaged in treatment. Moreover, inpatient care is not covered. Also, available studies from Sweden have seldom explored what and how much treatment is provided. This study, the first of its kind in Sweden in recent years, aims to examine treatment use (outpatient and inpatient) among all patients who during 2011 to 2021 had at least 1 contact with the Maria Ungdom Clinic (MU) in Stockholm. Compared to most previous research in this area, this large-scale study relies on routinely collected electronic health records and as such overcomes challenges related to self-reports (eg, recall bias) and under-coverage of certain populations in treatment studies.^5^ The study focuses on treatment provision across different year cohorts and according to age at first contact with MU, as well as potential differences according to sex and treatment type (outpatient and inpatient). Given the shortage of studies of this kind, the study adopts an explorative approach.

## Previous Research

There is a substantial literature on ASU. However, given the life phase adolescents are in, societal approaches to ASU are overall more focussed on prevention than on treatment.^6^ This imbalance is reflected in both research and monitoring efforts. For example, although many countries monitor trends in substance use in the general adolescent population, corresponding approaches are rarer in ASU populations. Monitoring efforts related to treatment, much of which come from the US, are typically based on larger community samples in which adolescents (and adults) are asked to self-report what kind of treatment they have received.^7-9^ These studies have estimated that only a small minority of those who according to self-reports meet criteria for a substance use disorder receive treatment, and a downward trend in self-reported cannabis use treatment has been observed for the past 2 decades for both adolescents and adults in the US.^7^

Comparatively little is known about adolescents in substance use treatment and what treatment they are offered and engage in. Much of the research has been carried out in the US, and calls for more research have recently been made with reference to the Nordic countries.^10^ Treatments developed for adults do not easily translate to adolescents,^6,11,12^ and adolescents and adults may differ on several variables that predict treatment entry, completion and success. For example, family and peer factors are often more important in ASU treatment,^13^ and adolescents typically do not present the type of withdrawal symptoms that are often observed among adults.^12^ Adolescents seldom seek treatment on their own accord and many are pressured into treatment.^13^ Consequently, they are less prone than adults to complete treatment.^14^ However, although the overall evidence base is somewhat scarce,^6^ some treatment approaches are more supported by research and policy than others, including cognitive behavioural therapy and motivational interviewing.^13^ While some harm reduction approaches have been discussed in relation to ASU treatment these are controversial in countries striving for total abstention,^13^ such as Sweden. This illustrates the important role of political factors in shaping the treatment system for substance use problems^15^ and of monitoring central treatment indicators across contexts.

Much ASU treatment research have explored characteristics of the patients and how they fare over time,^16-25^ and there are several qualitative studies on how adolescents experience the treatment.^1,26-28^ A recurring finding is that many patients have parallel mental health and criminal problems and that a larger share are males.^3,16-18,20^ Yet, once in treatment, females have been shown to attend more treatment sessions than males,^13^ which of course does not necessary mean that they have better outcomes. However, a recent systematic review of close to 100 studies found very few significant associations between characteristics such as age group, gender, and treatment setting and completion rates in adolescents and young adults.^14^ This points to the need for more research on, for example, who attends more treatment sessions or stays longer in treatment.

Missing in prior research is also a more extended temporal perspective, with most longitudinal studies covering a shorter time span, typically relying on self-reports and relatively small samples. We know comparably little about changes over longer periods of time among adolescents who are in ASU treatment. While there are some descriptive reports on temporal trends among patients in specialised municipal ASU treatment in Sweden, they focus more on the characteristics of the patients than what treatment they are provided and they do not include regional healthcare interventions.^2^

## Theoretical and Methodological Considerations

The study is aligned with a systems approach to substance use treatment.^29-31^ Research in this tradition takes a broader approach to treatment than what more demarcated efficacy or effectiveness studies typically do. This research does not consider outcome studies unimportant, but emphasises a range of other issues as equally crucial, including the broader socio-ecology and the various political factors that shape the system.^29,31^ In Sweden, substance use problems are managed by both municipal social services and regional healthcare, and the Maria clinics are located within an intricate professional network comprising many agencies (eg, child welfare, child and adolescent psychiatry, law enforcement, the educational system), and are run by either regional healthcare, municipal social services, or both in collaboration. Given the Swedish prohibitionist approach to all illicit drugs, ASU treatment focuses on total abstinence. Abstinence is frequently assessed by urine tests,^1^ and these controlling elements are to be combined with efforts to build rapport with the patients to improve treatment outcomes. The provision of treatment at Maria clinics is indeed a dynamic, complex practice that hardly can be captured by any single methodological approach.^32^ According to the widely influential Behavioural Model of Health Services Use,^33^ healthcare utilisation is a function of a range of “predisposing” (eg, demographics, attitudes towards services) and “enabling” (eg, organisational) factors, as well as perceived and professionally assessed needs, including also feedback loops between different components of the model. Thus, while the current study provides a comprehensive take on the treatment at Sweden’s largest ASU provider, this only covers parts of the complex clinical practices and the factors that shape how the treatment is provided.

## Data and Methods

### Sample

The study uses data on all patients who had a registered contact with Maria Ungdom (MU) in the Stockholm (capital) region of Sweden during 2011 and 2021, including both outpatient and inpatient treatment (n = 31 504). Some patients may have been in contact with MU in earlier periods but we do not have data on the extent to which this was the case. MU Stockholm is according to its homepage (www.mariaungdom.se/vara-mottagningar/hitta-mottagning) currently organised as 27 treatment clinics (“mottagningar”) in the Stockholm region: an inpatient ward, a few larger central outpatient clinics, as well as several smaller, local clinics that may only be staffed a few days a week. The patients may come in contact with the clinics in a number of different ways. For example, they may be referred to outpatient treatment at MU by the social services, their school, child and adolescent psychiatry, by parental referral, or their first contact may be due to intoxication. All patients who first receive inpatient treatment are also offered outpatient treatment. Some patients were excluded from analyses because of apparent errors in the health records related to their birth dates. As most MU clinics cater to people from age 13 up to the age of 20, and some up to the age of 25, we excluded all patients who were recorded to be 26 years or older (n = 1117) at first contact with MU. We also excluded all those who according to the case files were 12 years or younger at the first contact (n = 416). A couple of individuals who had their first contact during 2010 were also removed (n = 2) as they were too few to represent an entire cohort (ie, 2010). Otherwise, there were no exclusion criteria. This left a sample of 29 967 patients. Some information was only available for patients in outpatient treatment, so the number of patients vary in different parts of the Results section. No a priori power calculation was conducted for the following reasons: we utilised the full available sample; there was no single statistical contrast of greatest importance to guide effect size calculations; and the large sample size (even when broken into smaller subgroups) was believed to result in detectable differences of sufficient magnitude to be of scientific and clinical importance.

The time-frame in question (2011-2021) was chosen partly because this was a period of organisational stability for MU: the modern inpatient ward (which includes emergency care) was created in 2010 and one of the larger outpatient clinics was formed in 2011. Although there have been minor changes to the exact organisation of the smaller clinics during the period – some opening and some closing, within-municipality location changes etc. – the overall organisation has remained the same, as has the treatment uptake responsibility for the entirety of Region Stockholm. No major changes in the electronic health records system have occurred either. In terms of treatment provision, MU Stockholm has seen an increase in number of employed psychologists, with the aim of increasing the availability of psychological treatments and ADHD assessments. As a result of the implementation of structured healthcare process maps – a psychiatry-wide initiative in Region Stockholm – greater attention is now paid to psychiatric comorbidity, although the impact of this on uptake statistics remains to be examined. There has however been no major change in what types of treatment (eg, motivational interviewing, CBT etc.) are offered. While MU has remained a relatively stable point of reference in the local ASU landscape, child and adolescent psychiatry (a different organisational arm of regional healthcare) as well as municipal social services in Stockholm have faced well-known challenges during the period in question, including increasing demand for treatment, organisational changes, high staff turnover and more. Towards the end of the period covered, it was clarified in policy that isolated instances of substance use are not necessarily grounds for transfer from child and adolescent psychiatry to MU, although this is not yet implemented fully in practice. Moreover, social services in general are increasingly moving towards providing structured, evidence-based interventions, which imply that social services are now tasked with some of the treatment work previously carried out by MU Stockholm.

### Variables

Variables were derived from raw electronic health records, systematically compiled using a structured database search query. Variables included number of visits to outpatient treatment, number of inpatient treatment episodes, patient cohort, age at first contact with MU, sex and treatment type.

Number of *outpatient visits* and *inpatient episodes* were recorded as count variables, referring to the entire observation period, with no information provided about the date for each of the visits/episodes. These variables were used as they were originally coded in the analyses.

We distinguished between 11 year *cohorts*, defined according to year of first contact with MU during the observed period. Patients having their first contact during 2011 were defined as being members of the 2011 cohort, those who had their first contact during 2012 were defined as being members of the 2012 cohort and so on. *Patient age at first contact* was calculated by subtracting patients’ birth dates from the date of the first contact, rounded to full years. In the analyses we combined the age groups between 21 and 25 years to avoid problems with sparse data in some subgroups.

*Sex* (female/male) was derived from patients’ personal number. As to *treatment type*, patients who had at least 1 visit to an outpatient MU clinic but who had never been admitted to inpatient treatment at MU were defined as being in *outpatient treatment only*. Patients with at least 1 visit to an outpatient MU clinic and at least 1 admission to inpatient care were defined as being in *both outpatient and inpatient treatment*. Those who did not have any visits to outpatient treatment but at least 1 admission to inpatient care were defined as in *inpatient treatment only*. Of note, electronic health records covered only region-run ASU clinics in question; visits to social services (not involving MU Clinics), visits to corresponding clinics in other Swedish regions, or visits to private clinics, are not included. The data thus offer a complete view of care provided by the MU Clinics, but is not comprehensive in the sense that additional ASU treatment may, for some patients, have been offered by other actors.

### Statistical Analyses

Descriptive and multivariate techniques were used to analyse the data. Percent and mean values were used to summarise single variables. Negative binomial regressions were used in the multivariate analyses as the outcome variables had a larger variance than mean.

Two sets of regressions were run for outpatient visits and inpatient episodes, respectively. The first set estimated cohort and age trends overall and for subgroups defined by gender and treatment type. To allow for separate trends for subgroups, we included an interaction term between the specific subgroup variable and year cohort for respective age group. As very few patients in the 2019 to 2021 cohorts received inpatient treatment only, the analyses of inpatient episodes excluded these cohorts.

The second set of regressions adjusted for the fact that different cohorts (and age groups) had different potential exposure to treatment. The exposure was the entire period covered for those entering treatment in 2011 at a young age and only a few days for those entering at the end of 2021. There were large differences across cohorts in the days between first and last outpatient visit, ranging from 485.6 days on average for the 2011 cohort to 56.1 days for the 2021 cohort. Those who were at a younger age at first contact also had a longer time between first and last outpatient visit, ranging from 514.4 days on average among those who were 13 years at first contact to 210.9 among those who were 21 to 25 years. Cohorts differed in time in inpatient care as well: the 2011 cohort had an average of 130.5 days between the first episode and the last discharge and 2021 cohort had 10.95 days. The average number of days between first episode and last discharge was 205.3 among those who were 13 years at first contact and 9.72 among those who were 21 to 25 years.

To account for this, we included *potential time-in-treatment* as an offset variable in the regressions. This was calculated as the number of days between the first outpatient visit respective first inpatient episode at MU, and the last outpatient visit respective last discharge from inpatient treatment. In brief, offsetting a variable means first taking its natural logarithm and then constraining its coefficient in the regression model to 1.^34^ As logarithms cannot be defined for zeros, we added a small constant (+0.5) to the 2 potential time-in-treatment variables before taking their logarithms so that also patients with only 1 outpatient treatment visit/inpatient episode (eg, 0 days between first and last outpatient visits) could be included. In contrast to the first set of regressions that estimate counts, these models estimate rates, that is, number of visits/episodes per time unit.^34^ Results were presented as incidence rate ratios (IRR).

Statistical analyses were run in Stata vers. 17.0. Stata’s negbin command was used for the regression models. The margins command was used for predicting mean values in the first sets of analyses, and the marginsplot command was used to show these estimates graphically.

## Results

### Descriptive Statistics

Descriptive statistics are shown in Table 1. Females constituted slightly less than 37% of all patients having their first contact with MU between 2011 and 2021. Three out of 4 patients received outpatient treatment only and the smallest share received inpatient treatment only (6.6%). There was a larger share of patients in outpatient treatment only among males (79.9% vs 66.9%). Conversely, a larger share among females received inpatient treatment, either solely or combined with outpatient treatment (33% vs 20.1%). A third of the females received inpatient treatment, and the corresponding share was one-fifth among males.

**Table 1.** Descriptive statistics.

	All		Females (n = 10 991)		Males (n = 18 976)	
	n	%	n	%	n	%
Treatment type						
Outpatient only	22517	75.14	7355	66.92	15162	79.90
Outpatient and inpatient	5468	18.25	2674	24.33	2794	14.72
Inpatient only	1982	6.61	962	8.75	1020	5.38
Age at first contact with MU						
Mean (std)^a^	29967	16.76 (2.42)	10991	16.72 (2.58)	18976	16.77 (2.32)
Median^a^		16		16		16
13	1414	4.72	694	6.31	720	3.79
14	3406	11.37	1529	13.91	1877	9.89
15	4910	16.38	1787	16.26	3123	16.46
16	5604	18.70	1773	16.13	3831	20.19
17	5599	18.68	1623	14.77	3976	20.95
18	3361	11.22	1266	11.52	2095	11.04
19	2526	8.43	1018	9.26	1508	7.95
20	780	2.60	344	3.13	436	2.30
21-25	2367	7.90	957	8.71	1410	7.43
Number of outpatient visits	27 985		10 029		17 956	
Mean (std)		12.31 (20.69)		12.37 (22.98)		12.27 (19.29)
Median		5		4		6
Number of days between first and last outpatient visit	27 985		10 029		17 956	
Mean (std)		307.57 (520.02)		292.66 (516.69)		315.90 (521.71)
Median		70		52		80
Number of inpatient episodes	7450		3636		3814	
Mean (std)		1.59 (1.87)		1.62 (2.17)		1.56 (1.54)
Median		1		1		1
Number of days between first inpatient episode and last inpatient discharge	7450		3636		3814	
Mean (std)		83.61 (267.59)		96.82 (305.23)		71.01 (225.26)
Median		1		1		1

a Age group 21 to 25 not combined.

On average, the patients were 16.8 years old when they had their first contact with MU, with a median age of 16 years. The figures were similar for females and males. Overall, the relatively most common age at first contact was 16 years, closely followed by 17 and 15 years, with some notable sex differences. A larger share of the females were 13 years or 14 years at the first contact, whereas a larger share of males were 16 or 17 years.

Outpatients had on average 12.3 visits to MU and the high standard deviation (20.7) indicated substantial heterogeneity in the number of visits. There were in total 344 388 outpatient visits recorded for the patients (not shown in Table 1). The median number of visits were 5. The average number of outpatient visits were similar across sexes, but with a somewhat larger variance among females. The coefficient of variation (ie, SD/M) was 1.86 for females and 1.57 for males. The mean number of days between first and last visit to MU was 307.6, with a substantial spread (std = 520). The median was 70 days. Males had a longer average time between first and last visit.

Inpatients had an average of 1.59 inpatient episodes, with only small sex differences. The median number of episodes was 1. The patients had a total of 11 824 inpatient episodes (not shown in Table 1). There was a large variation in the number of inpatient episodes, particularly among females. The coefficient of variation was 1.34 for females and 0.99 for males. The mean number of days between the first inpatient event and the last discharge was 83.61 days, with a large standard deviation and the median was 1 day. A longer time had passed between first inpatient episode and last discharge among females.

### Cohort and Age Trends

#### First Contacts

Table 2 presents the number of patients who had their first contact with MU at a given calendar year between 2011 and 2021. Overall, the distribution over the years was similar, with a somewhat larger share having their first contact during 2011 and a somewhat smaller share had their first contact during 2021. The sex distribution fluctuated across cohorts, ranging from 30.73% females for the 2017 cohort to 44.12% for the 2021 cohort. The average age at first contact was very similar across cohorts, and this was the case among both females and males.

**Table 2.** Year at first contact with Maria clinic (outpatient and inpatient), all and by gender.

	All				Females				Males			
	n	%	Age (*M*, std)		n	%	Age (*M*, std)		n	%	Age (*M*, std)	
2011	3928	13.11	16.98	2.08	1486	37.83	17.06	2.31	2442	62.17	16.93	1.92
2012	2751	9.18	16.91	2.22	1017	36.97	16.98	2.39	1734	63.03	16.88	2.12
2013	2695	8.99	16.89	2.42	965	35.81	16.96	2.63	1730	64.19	16.85	2.30
2014	2402	8.02	16.77	2.53	946	39.38	16.75	2.71	1456	60.62	16.78	2.41
2015	2447	8.17	16.89	2.60	898	36.70	16.93	2.69	1549	63.30	16.87	2.54
2016	2429	8.11	16.97	2.53	852	35.08	17.00	2.76	1577	64.92	16.95	2.40
2017	2737	9.13	16.77	2.49	841	30.73	16.82	2.72	1896	69.27	16.75	2.38
2018	2812	9.38	16.69	2.40	920	32.72	16.65	2.63	1892	67.28	16.70	2.29
2019	2875	9.59	16.41	2.35	981	34.12	16.38	2.47	1894	65.88	16.43	2.29
2020	2656	8.86	16.39	2.43	1099	41.38	16.17	2.44	1557	58.62	16.55	2.41
2021	2235	7.46	16.56	2.65	986	44.12	16.22	2.57	1249	55.88	16.83	2.67

Abbreviations: *M*, mean; std, standard deviation.

#### Outpatient Treatment

**Figure 1.**
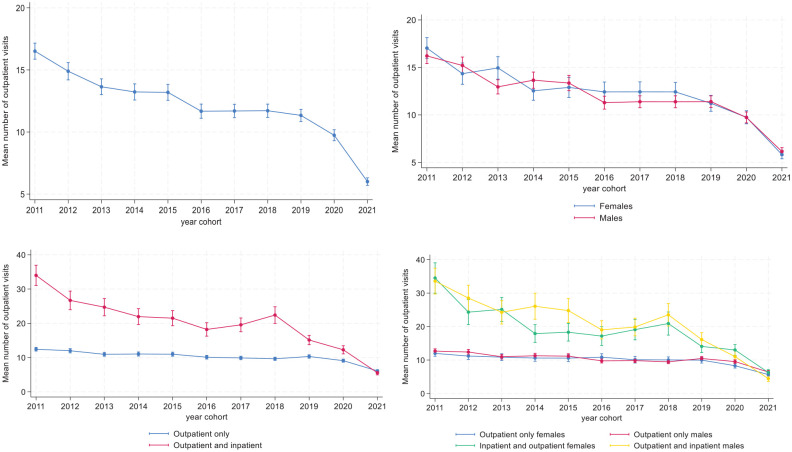
(a-d). Mean number of outpatient visits to MU according to year cohort, all (upper left) by sex (upper right), treatment type (lower left) and by sex and treatment type (lower right). Predicted means with 95% confidence intervals. Estimates are derived from a negative binomial regression. The estimates in the upper left panel include cohort group as a predictor and the other estimates also include the focal predictor and its interaction with cohort group. N = 27 985.

Figure 1a to d show the mean number of visits to outpatient treatment for the year cohorts. There was a decline in visits from earlier to later cohorts, and this was evident for both females and males. However, there were differential patterns for those who were in outpatient treatment only and those who also were in inpatient treatment. The number of visits for outpatient and inpatient patients declined across cohorts, but this was not the case among patients in outpatient treatment only. In fact, while 2011 cohort patients who received both outpatient and inpatient treatment on average had over 20 more visits than those who received outpatient treatment only, no such differential was observed between these group in the 2021 cohort.

**Figure 2.**
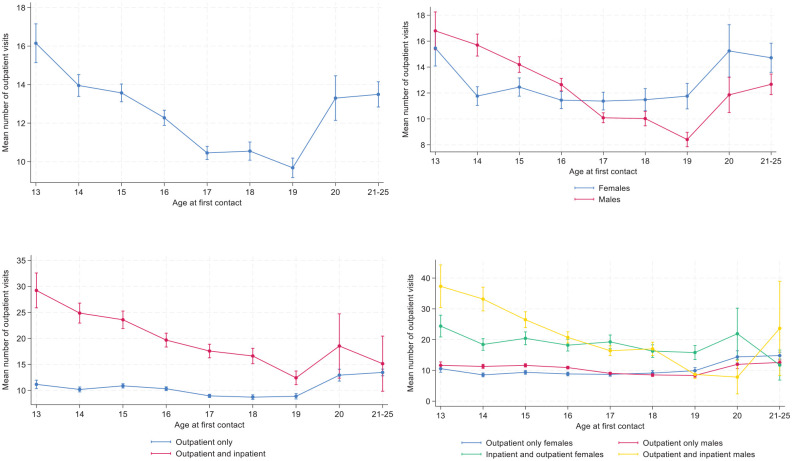
(a-d). Mean number of outpatient visits to MU according to age at first contact with MU, all (upper left) by sex (upper right), treatment type (lower left) and by sex and treatment type (lower right). Predicted means with 95% confidence intervals. Estimates are derived from a negative binomial regression. The estimates in the upper left panel include age at first contact with MU as a predictor and the other estimates also include the focal predictor and its interaction with age. N = 27 985.

Figure 2a to d present the mean number of visits to outpatient treatment by age at first contact with MU (regardless of cohort). There was a downward trend from being 13 years at first contact to being 19 years old; the former on average had about 16 visits and the latter about 10 (Figure 2a). From 19 years of age to the oldest age group (21-25 years), there was again an increase in the number of visits. The patterns were relatively similar for the sexes (Figure 2b), but differed between treatment types (Figure 2c). Note, though, the large confidence intervals for some of the older age groups among patients in both outpatient and inpatient treatment. The most obvious differences according to age at first contact was observed for males in both outpatient and inpatient treatment (Figure 2d).

Table 3 presents results from negative binomial regressions of number of outpatient visits on year cohort, age at first contact, sex and treatment type. All models were adjusted for differences in potential exposure time to treatment. Model 1 only includes year cohort, and shows in contrast to the results in Figure 1a a positive association between year cohort and outpatient visits; the rate of outpatient visits was higher the later the year cohort. The 2021 cohort had more than twice as high a rate of outpatient visits than the 2011 cohort. Model 2 shows the estimates for age at first contact with MU. Opposite to the analyses that did not adjust for exposure time (Figure 2a), the rate of outpatient visits increased between age 13 at first contact to age 19, where the latter had a rate that was more than twice as high as the former. Those who were 20 or 21 to 25 years of age at first contact also had a higher rate of outpatient visits compared to those who were younger at first contact. The associations for both year cohort and age at first contact were similar, but were strengthened, when both were included in the same model (model 3) and when also adjusting for sex and treatment type (model 4). In the fully adjusted model, the rate of outpatient visits was lower among those in both outpatient and inpatient treatment compared to those in outpatient treatment only (IRR = 0.91 [95% CI = 0.88-0.94]) and it was also lower among males than among females (IRR = 0.85 [95% CI = 0.82-0.87]).

**Table 3.** Negative binomial regression for the association between year cohort, age at first contact, treatment type and sex and number of outpatient visits to MU.

	Model 1		Model 2		Model 3		Model 4	
	IRR	95% CI	IRR	95% CI	IRR	95% CI	IRR	95% CI
Year cohort								
2011 (ref)	1				1		1	
2012	1.09	1.03-1.16			1.12	1.06-1.19	1.12	1.05-1.18
2013	1.07	1.01-1.13			1.12	1.06-1.19	1.12	1.06-1.19
2014	1.14	1.08-1.22			1.22	1.15-1.29	1.21	1.14-1.28
2015	1.16	1.09-1.23			1.23	1.15-1.30	1.22	1.15-1.30
2016	1.22	1.14-1.29			1.27	1.20-1.35	1.28	1.20-1.35
2017	1.25	1.18-1.32			1.33	1.26-1.41	1.34	1.26-1.42
2018	1.25	1.18-1.33			1.34	1.27-1.42	1.34	1.27-1.42
2019	1.34	1.27-1.42			1.48	1.40-1.56	1.48	1.40-1.56
2020	1.57	1.49-1.66			1.76	1.67-1.87	1.74	1.65-1.84
2021	2.26	2.12-2.40			2.54	2.39-2.69	2.49	2.34-2.64
Age								
13 (ref)			1		1		1	
14			1.11	1.03-1.19	1.14	1.07-1.22	1.15	1.07-1.23
15			1.19	1.11-1.27	1.27	1.19-1.35	1.28	1.20-1.37
16			1.30	1.21-1.39	1.41	1.32-1.50	1.44	1.35-1.53
17			1.42	1.32-1.51	1.58	1.48-1.68	1.61	1.51-1.72
18			1.76	1.63-1.90	1.99	1.85-2.14	2.02	1.88-2.17
19			2.10	1.93-2.28	2.39	2.21-2.59	2.42	2.23-2.62
20			1.70	1.53-1.88	1.89	1.71-2.09	1.86	1.69-2.06
21-25			1.55	1.44-1.67	1.69	1.57-1.82	1.67	1.55-1.80
Outpatient (ref)							1	
Inpatient & outpatient							0.91	0.88-0.94
Female (ref)							1	
Male							0.85	0.82-0.87
Days between first and last visit (offset)	1		1		1		1	

N = 27 985.

#### Inpatient Treatment

**Figure 3.**
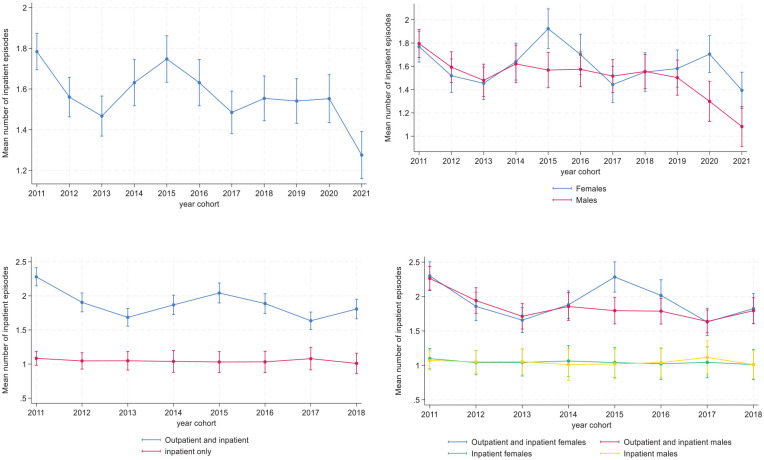
(a-d). Mean number of inpatient episodes at MU according to year cohort, all (upper left) by sex (upper right), treatment type (lower left) and by sex and treatment type (lower right). Predicted means with 95% confidence intervals. Estimates are derived from a negative binomial regression. The estimates in the upper left panel include cohort group as a predictor and the other estimates also include the focal predictor and its interaction with cohort group. The lowermost figures only include cohort 2011 to 2018 because of sparse cells for inpatient patients only among cohorts 2019 to 2021. N = 7450 for uppermost figures and 5843 for lowermost figures.

The mean number of care episodes in inpatient treatment according to year cohort are shown in Figure 3a to d. While the first cohort had a larger number of care episodes than the last, the relation was non-linear across several intermediate cohorts, with a peak for the 2015 cohort (Figure 3a). This peak was mainly observed for females (Figure 3b) and for patients in both outpatient and inpatient treatment (Figure 3c), and was particularly evident among females in both outpatient and inpatient treatment (Figure 3d).

**Figure 4.**
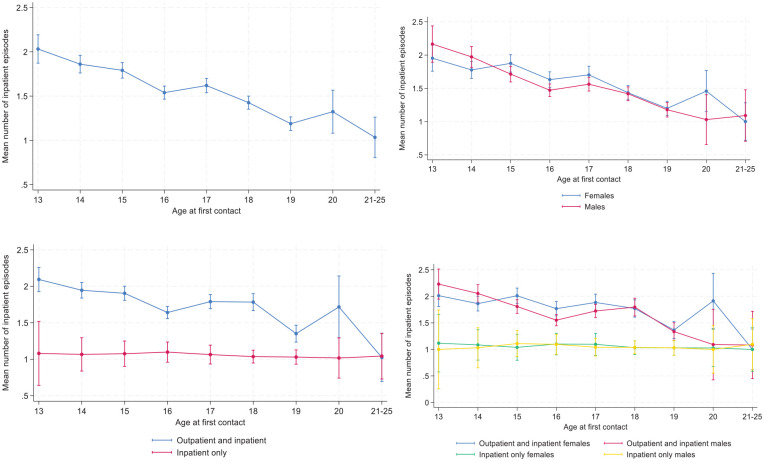
(a-d). Mean number of inpatient episodes at MU according to age at first contact with MU, all (upper left) by sex (upper right), treatment type (lower left) and by sex and treatment type (lower right). Predicted means with 95% confidence intervals. Estimates are derived from a negative binomial regression. The estimates in the upper left panel include age at first contact with MU as a predictor and the other estimates also include the focal predictor and its interaction with age. N = 7450.

There was a negative association between number of inpatient episodes and age, such that those who were older had a larger number of inpatient episodes (Figure 4a). The patterns were similar for the sexes (Figure 4b) but differed between those in outpatient and inpatient treatment and those in inpatient treatment only (Figure 4c). In the latter group, the number of inpatient episodes were essentially the same regardless of age at first contact.

Table 4 shows the results from negative binomial regressions of number of inpatient episodes on year cohort, age at first contact, gender and treatment type (outpatient and inpatient vs inpatient only). The regression models were adjusted for differences in potential exposure time. In these analyses, there was no clear pattern between year cohort and inpatient episode rates, and the confidence intervals for the 2016 and 2017 cohorts overlapped 1 (model 1). There was a clear gradient for age at first contact where those who were older at first contact had a higher estimated inpatient episode rate (model 2). Thus, when adjusting for exposure time, the age pattern shown in Figure 4a was reversed. The estimates for year cohort were similar when adjusting for age at first contact, and the estimates for the latter were similar when adjusting for year cohort (model 3). However, in the fully adjusted model, the IRRs increased for some year cohorts, and none of the cohort year indicators overlapped 1. In contrast, when adjusting for sex and treatment type, the IRRs decreased for most age groups.

**Table 4.** Negative binomial regression for the association between year cohort, age at first contact, treatment type and sex and number of inpatient events to MU. Due to sparse cells, only year cohorts 2011 to 2018 are included. N = 5843.

	Model 1		Model 2		Model 3		Model 4	
	IRR	95% CI	IRR	95% CI	IRR	95% CI	IRR	95% CI
Year cohort								
2011 (ref)	1				1		1	
2012	1.26	1.07-1.50			1.25	1.05-1.48	1.33	1.13-1.57
2013	1.39	1.17-1.66			1.35	1.13-1.60	1.59	1.34-1.88
2014	1.21	1.01-1.45			1.22	1.02-1.47	1.47	1.23-1.76
2015	1.24	1.04-1.49			1.28	1.07-1.53	1.52	1.28-1.81
2016	1.11	0.92-1.34			1.12	0.93-1.34	1.31	1.09-1.57
2017	1.09	0.91-1.30			1.12	0.93-1.34	1.36	1.14-1.62
2018	1.23	1.02-1.47			1.25	1.04-1.50	1.47	1.23-1.75
Age								
13 (ref)			1		1		1	
14			1.39	1.06-1.83	1.39	1.06-1.82	1.37	1.04-1.79
15			1.47	1.13-1.92	1.48	1.14-1.93	1.40	1.07-1.81
16			2.05	1.59-2.65	2.07	1.60-2.67	1.90	1.48-2.46
17			2.10	1.62-2.72	2.13	1.64-2.77	1.82	1.40-2.36
18			2.95	2.28-3.82	2.95	2.28-3.83	1.76	1.36-2.29
19			3.40	2.61-4.43	3.40	2.61-4.43	2.03	1.55-2.65
20			3.72	2.39-5.79	3.66	2.35-5.70	1.89	1.22-2.95
21-25			3.73	2.32-6.00	3.61	2.25-5.82	2.03	1.26-3.26
Outpatient and inpatient (ref)							1	
Inpatient							2.72	2.44-3.03
Female (ref)							1	
Male							0.88	0.80-0.97
Days between first episode and last discharge (offset)	1		1		1		1	

## Discussion

System monitoring is central in substance use treatment research.^29^ While there are systematic efforts to monitor treatment demand based on patient characteristics,^35^ little is known about what treatment adolescents receive. Informed by a systems approach to substance use treatment,^29-31^ and acknowledging that the complexities of clinical practice are difficult to fully capture in research,^32^ this study explored the amount and type of treatment received according to year cohort and age at first contact with Maria Ungdom Stockholm, Sweden’s largest regional provider of ASU treatment.

Reflecting prior research, the majority of the patients were males,^17^ although the gender distribution converged somewhat in the last 2 cohorts. The most common treatment was outpatient only, which comprised about 75% of all patients. However, a larger share of males than females received outpatient treatment only (80% vs 67%) and the reverse held true for inpatient treatment only as well as both outpatient and inpatient treatment. Thus, female patients overall seemed to receive somewhat more comprehensive treatment. There were only marginal sex differences in the average age at first contact with MU, but females were more likely to enter treatment at 13 years or 14 years, whereas males were more likely to enter at the more typical age of 16 and 17 years. Females in ASU treatment overall have more extensive problems than males, including having both an earlier substance use debut^20^ and more severe substance use problems.^17,19^ They have also a higher prevalence of mental health problems.^20,24^ Our findings of sex differences in type of treatment received may reflect such differences.

On average, patients in outpatient treatment had about 12 visits to a clinic, with no evident sex differences. However, there was a large variation in the number of outpatient visits and this was particularly evident among females. There was notable variation in number of inpatient episodes as well, and the variation was more apparent among females. These findings agree with prior studies showing that patients at Maria clinics are a highly heterogenous group.^3^ Other research has also confirmed heterogeneity in substance use treatment trajectories among young patients.^4^

We found differential trends for outpatient visits and inpatient episodes according to year cohort. The analyses that did not adjust for potential time in treatment showed a clear downward trend from about an average of 16 outpatient visits for the 2011 cohort to about 6 visits for the 2021 cohort. For inpatient care episodes, the results were less clear-cut. Even though the 2011 cohort received more inpatient episodes than the 2021 cohort, there were fluctuations across intermediate cohorts. We also identified disparate trends in treatment use for the different treatment types. The number of outpatient visits was relatively stable across cohorts among outpatients only, but it sharply declined among patients who also received inpatient treatment, and this seemed to drive the overall, downward trend in outpatient visits. The complex pattern in inpatient episodes across cohorts was also more obvious among patients who received both outpatient and inpatient treatment, while there were essentially no cohort differences among those in inpatient treatment only. This points to the importance of separating different treatment types in studies on adolescent treatment use, something that is not always done in smaller studies.^4^ Interestingly, females in both outpatient and inpatient treatment had the most varying trend across year cohorts in the number of inpatient care episodes. This underscores that heterogeneity not only exists in relation to patient characteristics^3^ or treatment trajectories,^4^ but also in relation to when different subgroups enter treatment.

When adjusting for differences in potential exposure time for treatment, the patterns in outpatient visits were reversed, with a higher outpatient visit rate for later year cohorts. Thus, per time unit, later cohorts had more visits, and this remained when adjusting for age at first contact and type of treatment. In the fully adjusted model, patients who had their first contact with MU during 2021 had an outpatient visit rate that was about 150% higher than patients who had their first contact during 2011. This apparent intensification of outpatient treatment among later cohorts is interesting and should be explored further. It is plausible that increased prevalence of cannabis, illegal in Sweden, as the primary drug at Maria clinics^2^ may have intensified treatment delivery, but changes in the social-ecology surrounding treatment,^31^ referrals from the social services and child and adolescent psychiatry and in clinical routines may also have played a role. For example, there may have been an increase in urine drug testing over time and the patient group may also have become more burdened in recent years. For example, prior estimates^2^ suggest that a larger share of adolescents in contact with Maria clinics had prior or ongoing contact with psychiatric services during the latter part of the time period covered, suggesting that later cohorts may have more complex needs. At the same time, while cannabis over time has become more common as the primary substance, there has been a decline in the frequency of use of the primary substance and in risky alcohol consumption, and there seems to be no clear time trends in criminal offences (such as minor drug offence).^2^ The decline in alcohol use is also reflected in a downward trend in diagnoses for harmful use or dependence of alcohol among all Swedish patients in specialised, regional substance use treatment in Sweden during this period.^36^ Likewise, national estimates suggest that there have been no obvious changes in the prevalence of cannabis and other illicit substance use among Swedish adolescents during the period covered, although there appears to have been a slight increase in the frequency of use among users.^37^ Reflecting the trend across a number of countries, alcohol consumption has dropped substantially in the Swedish adolescent population during this period.^37^

Thus, we find it unlikely that the differences across cohorts would be due only to different patient needs. Further research may take a deeper look into variations in clinical routines among ASU treatment providers over time for understanding temporal trends in treatment provision. Attending to the emergence and dynamics of organisational routines are crucial for understanding how organisations work,^38^ and may give important clues to variations across time and patients in treatment. A strength of the current study, however, is that Maria Ungdom Stockholm has been around since the 1960s,^23^ with a relatively fixed role in an otherwise changing healthcare organisational landscape. The more notable changes at Maria Ungdom occurred before or during the first year of the period (the forming of the modern inpatient ward in 2010 and one of the larger outpatient clinics in 2011). Thus, the trends identified here are unlikely to be due to any profound within-organisational changes at Maria Ungdom Stockholm during the period.

For inpatient episodes, the analyses that adjusted for different exposure time among the year cohorts presented a less clear pattern than what was the case for outpatient visits. Although all subsequent year cohorts had a higher inpatient episode rate than the 2011 cohort, the pattern was not linear. The largest difference in inpatient episode rates was found between the 2015 cohort and the 2011 cohort, also in the fully adjusted model. It is difficult to know why there was only a clear trend for outpatient visits, but the large differences in inpatient episodes between the 2015 and the 2011 cohort may in part be due to the large inflow of immigrants during the 2015 “refugee crisis,” which resulted in an increase in the number of unaccompanied children being admitted to inpatient treatment. Of note, the number of inpatient episodes related to dependence or harmful illicit drug use also peaked in 2015 among all Swedish individuals in regional healthcare during this period, with the highest figures found for Stockholm.^36^

The average age at first contact with MU was stable across cohorts; there was thus little to suggest that patients have become younger over time. Regarding age at first contact, the analyses that did not consider potential exposure time to treatment showed a clear decrease in the number of visits in age groups 13 to 19, which then increased again for age groups 20 and 21 to 25 years. This finding broadly agrees with a Norwegian study^4^ showing that younger age is associated with more treatment use. This overall decrease over age groups, however, was mainly observed among males and among those in both outpatient and inpatient treatment.

There was also a lower number of inpatient episodes among those who were older at first contact. In this case as well, the trends differed according to treatment type. There was a negative association between age and number of inpatient episodes among patients in both inpatient and outpatient treatment, but there were essentially no differences between age at first contact among those in inpatient treatment only. Similar to outpatient visits, there were pronounced differences in number of inpatient episodes according to treatment type at lower ages at first contact (higher number of episodes among those in both outpatient and inpatient treatment), but this gap diminished substantially among those who were older at first contact.

When adjusting for potential exposure time to treatment, the association between age at first contact and number of outpatient visits changed direction. In these analyses, there was a positive association between age at first contact and number of outpatient visits in age groups 13 to 19; the rate was more than twice as high in those aged 19 at first contact compared to those who were 13 years old. A similar pattern emerged for number of inpatient episodes, where the difference between those who were 13 years at first contact and some older age groups were substantial. For example, those who were 19 years old at first contact had twice the rate of inpatient episodes compared to those who were 13 years old at first contact. Thus, whereas those who were older at first contact overall received less outpatient and inpatient treatment, they were provided more treatment per time unit.

### Strengths and Limitations

Some limitations should be noted. First, the data could potentially include systematic errors in recording,^39^ and there could potentially be variations in recording across staff and facilities^4^ as well as over time. However, it is unlikely that staff would forget or neglect to record information about outpatient and inpatient treatment as such recording is a legal requirement in Sweden. Second, as our data do not include visits to social services (not involving municipal MU Clinics), to corresponding clinics in other regions in Sweden, or visits to private clinics, our study does not cover all treatment provided to adolescents. Third, we lack data on when each of the outpatient visits and impatient episodes occurred for those who had more than 1 visit/episode; the data only include information on the first and last outpatient visit/inpatient episodes and the total number of visits/episodes for each patient. As a consequence of this, our approach for adjusting for different potential exposure time to treatment was somewhat crude. Also, the study is limited to the Stockholm region which may differ in important respects from the specialised treatment provided in other parts of Sweden. From a complexity perspective, this is indeed to be expected.^32^ The fact that the study only covered regional healthcare should be considered as well. No a priori power calculation is also a limitation.

Key strengths include total coverage of patients enrolled at Sweden’s largest provider of specialised ASU treatment. Employing objective measures of treatment use, we also did not face problems related to self-reports and non-response. The large sample allowed for separate analyses of outpatient and inpatient treatment, and for separate subgroups of patients. The longer time period should also be considered a strength, as much prior research covers only shorter time spans. Also, compared to many other studies assessing treatment use over time, the study relied on cohort data and not repeated cross-sectional surveys which have often been the case when assessing treatment use.^8^

## Conclusion

Between 2011 and 2021, there were close to 345 000 outpatient visits and nearly 12 000 inpatient episodes at Maria Ungdom Stockholm in Sweden. This study on patients at Sweden’s largest ASU clinic between 2011 and 2021 revealed that earlier cohorts and those who were younger at first contact received more outpatient treatment and the same pattern was found for age at first contact and inpatient treatment. The treatment rate was higher for both outpatient and inpatient treatment among later cohorts and patients who were at an older age at first contact, suggesting that these groups were provided with more intensive treatment.
